# Effects of Laser-Beam Defocus on Microstructural Features of Compositionally Graded WC/Co-Alloy Composites Additively Manufactured by Multi-Beam Laser Directed Energy Deposition

**DOI:** 10.1038/s41598-020-65429-8

**Published:** 2020-06-02

**Authors:** Takahiro Kunimine, Ryusei Miyazaki, Yorihiro Yamashita, Yoshinori Funada

**Affiliations:** 1 Faculty of Mechanical Engineering, Institute of Science and Engineering, Kanazawa University, Kakuma-machi, Kanazawa, Ishikawa, 920-1192 Japan; 2 Division of Mechanical Science and Engineering, Graduate School of Natural Science and Technology, Kanazawa University, Kakuma-machi, Kanazawa, Ishikawa, 920-1192 Japan; 3 Industrial Research Institute of Ishikawa, Kuratsuki 2-1, Kanazawa, Ishikawa, 920-8203 Japan

**Keywords:** Engineering, Materials science

## Abstract

Establishing processing routes for obtaining metal-matrix composites (MMCs) with uniformly-dispersed reinforcements is one of the main subjects in additively manufactured composite materials to achieve designed microstructures and mechanical properties. Here we report on the microstructural features of compositionally graded WC/Co-alloy composites additively manufactured by multi-beam laser directed energy deposition (multi-beam LDED). For tailoring microstructures of compositionally graded WC/Co-alloy composites with uniformly-dispersed reinforcements, the combinational method: the laser-beam defocus function in the multi-beam LDED system and granulated powder was attempted. By laser defocusing in the multi-beam LDED system, composites with uniformly-dispersed WC particles in Co alloy matrix was successfully obtained due to melting of Co bond in WC-12 wt.%Co granulated particles. It was found that the laser defocusing of multi-beam lasers affects temperature increase of flying powder during the laser focusing area, resulting in change of processing mode from melt-pool mode to thermal spray mode. The preferable property gradients in the WC/Co-alloy composites could be obtained by controlling the feeding rate of the powders and laser-beam defocus. These experimental results demonstrated the effectiveness of the laser-beam-defocus function in the multi-beam LDED system as a key factor for tailoring microstructures of additively-manufactured functionally graded MMCs with uniformly-dispersed reinforcements.

## Introduction

### Multi-beam laser directed energy deposition (Multi-Beam LDED)

Laser directed energy deposition (LDED) is one of the additive manufacturing (AM) process^1^. In the LDED process, blown powder is subjected to *in-situ* laser melting^1,2^. Compared with powder bed fusion AM process, the LDED process has advantages in powder handling in terms of used amount of powders, powder replacement, and controlling of multiple kinds of powders. The LDED process is usually applied to engineered components. Especially, the LDED process has been utilized for coating of layer-materials with wear and corrosion resistance on base materials such as dies and tools^3^, or repairing engineered components and products such as turbine blades^4–6^. In addition, processing of compositionally graded materials can be realized by effectively leveraging the LDED system with using multiple powder feeders.

**Figure 1:**
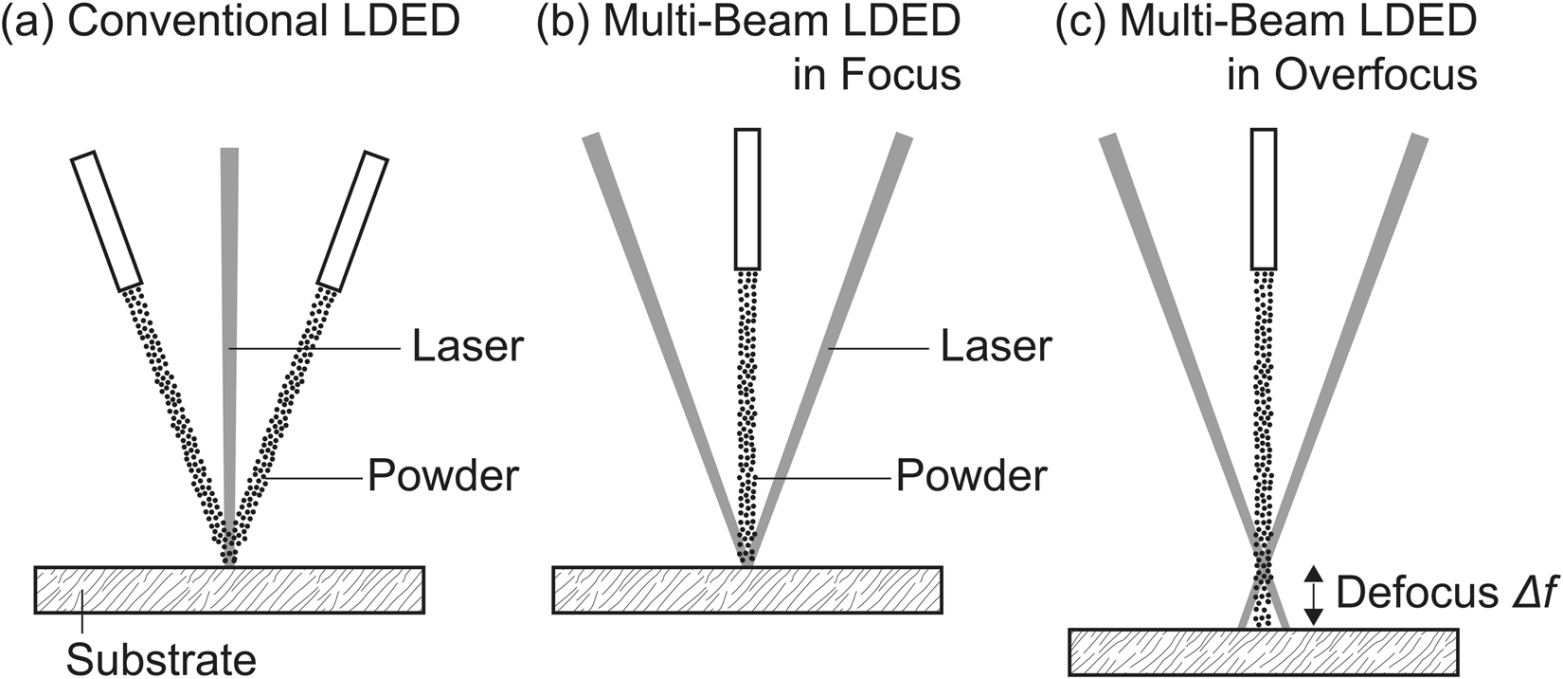
Schematic illustrations showing laser geometry in (**a**) a conventional single-beam laser directed energy deposition (conventional LDED) and a state-of-the-art multi-beam laser directed energy deposition (multi-beam LDED) in (**b**) focus and (**c**) overfocus of multi lasers on the surface of the substrate.

Figure 1(a) to (c) shows schematic illustrations showing laser geometry in (a) a conventional single-beam laser directed energy deposition (conventional LDED) and a state-of-the-art multi-beam laser directed energy deposition (multi-beam LDED)^7^ in (b) focus and (c) overfocus of multi lasers on the surface of the substrate. In the conventional LDED system, laser beam is vertically provided on the substrate followed by laser melting of substrate material^1^. The cladding layer is made by providing powders laterally into laser-melt pool as shown in Fig. 1(a). In contrast to the conventional LDED system, six oblique-incident lasers can be focused on the surface of the substrate in the multi-beam LDED system^7^ used in this study as shown in Fig. 1(b). Powder is vertically provided on the substrate from the powder injection nozzle followed by laser melting. This laser geometry provide not only more stable powder-feeding but also new functionality: laser-defocus function using the focused six lasers by changing the substrate position as shown in Fig. 1(c). By changing the substrate position, the focused six-lasers directly heat the powder stream. Then, the heated melt or semi-melt powder is deposited on the substrate. Therefore, AM process in the multi-beam LDED system may change from melt-pool mode to thermal spray mode by changing the degree of laser defocus *Δf*. However, little is known about the effects of laser-beam defocus on microstructural features of additively manufactured materials by the multi-beam LDED.

### Additive manufacturing of compositionally graded metal-matrix composites (MMCs) by the LDED process

**Figure 2:**
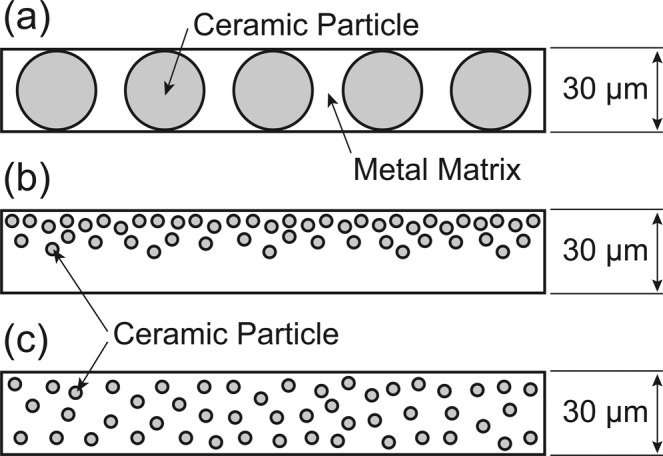
Schematic illustrations showing single-layers of MMCs with the thickness of 30 μm, having (**a**) large ceramic particles, (**b**) ununiformly and (**c**) uniformly dispersed small ceramic particles, processed by the LDED process.

Ceramic coatings applied to metallic components are intended to enhance the material properties such as heat, corrosion, and wear resistances^8^. However, crack initiation and propagation usually occurs in a ceramic coating layer by thermal stress due to the difference of thermal expansion coefficient between the ceramic and metallic materials. Application of metal-matrix composites (MMCs) based functionally graded materials (FGMs)^9^ for coatings is the most promising way to overcome this problem by smoothing the thermal stress distribution across the layers. Although various processing techniques for FGMs were developed^9^ based on casting^10,11^, spark plasma sintering^12^ and so on, these processing techniques are generally applicable to products with limited shapes and materials, and time-consuming processes. The LDED process is expected to realize the rapid processing of FGMs and broaden the applicable products. However, little is known about the MMCs processing by the LDED so far. Especially, establishing processing routes for obtaining MMCs with uniformly-dispersed reinforcements for a single-layer is one of the main subjects in the field of additively manufactured MMCs-based FGMs to achieve designed microstructures and mechanical properties. In AM of MMCs by the LDED process, two kinds of powders: metallic and ceramic powders are used at least. Figure 2(a) to (c) shows single-layers of MMCs with the thickness of 30 μm, having (a) large ceramic particles, (b) ununiformly and (c) uniformly dispersed small ceramic particles, processed by the LDED. For laser beam melting, metallic powders having particle-size ranging from about 15 μm to 150 μm are generally used^13^. Basically, coarse powder with size of about 100 μm to 150 μm is used in powder fed techniques, whereas fine powder with size of about 10 μm to 45 μm is used in powder bed techniques. For ceramic particles, there is no limitation for optimal particle size in terms of laser melting since ceramic particles are used for reinforcement. However, ceramic particles larger than a single-layer thickness cannot be used as shown in Fig. 2(a). As the hardness of ceramics generally follows a Hall-Petch-like relationship^14^, it is important to use finer ceramic particles in order to obtain finer microstructure with higher hardness. However, there is a few hurdles in controlling particles with nano to submicron scale in the LDED system. First, it should be difficult to obtain a deposited MMCs layer with uniformly dispersed small ceramic particles as shown in Fig. 2(c) due to the difference of mass density between ceramic and metallic particles. An inhomogeneous microstructure as shown in Fig. 2(b) should be obtained, especially for the use of nanoparticles. Second, powder controlling during powder feeding in the LDED system becomes significantly difficult with decreasing the particle size due to agglomeration of powders, electrostatic force, and so on. Although Doñate-Buendía *et al*. conducted the study for submicron-sized oxide dispersion-strengthened alloys by the LDED, the volume fraction of ceramics was low^15^. Our strategy for tailoring microstructures of MMCs with uniformly-dispersed reinforcements is based on the combinational method: the laser-beam defocus function in the multi-beam LDED system and granulated powder. Use of granulated powder provides a solution for these problems as a key factor to obtain finer microstructure of MMCs.

In this study, AM process of compositionally graded MMCs on substrate by the multi-beam LDED system was investigated. Compositionally graded tungsten carbide (WC)/Co-alloy composites coating on steel was attempted for the case intended to add wear resistance to press-punching die for automotive industry. Especially, effects of laser-beam defocus *Δf* of the multi-beam LDED system on microstructural features of additively manufactured MMCs were focused on, to investigate the feasibility of this function as key factors for tailoring microstructure of MMCs with the use of granulated powder.

## Materials and Methods

### Materials

**Figure 3:**
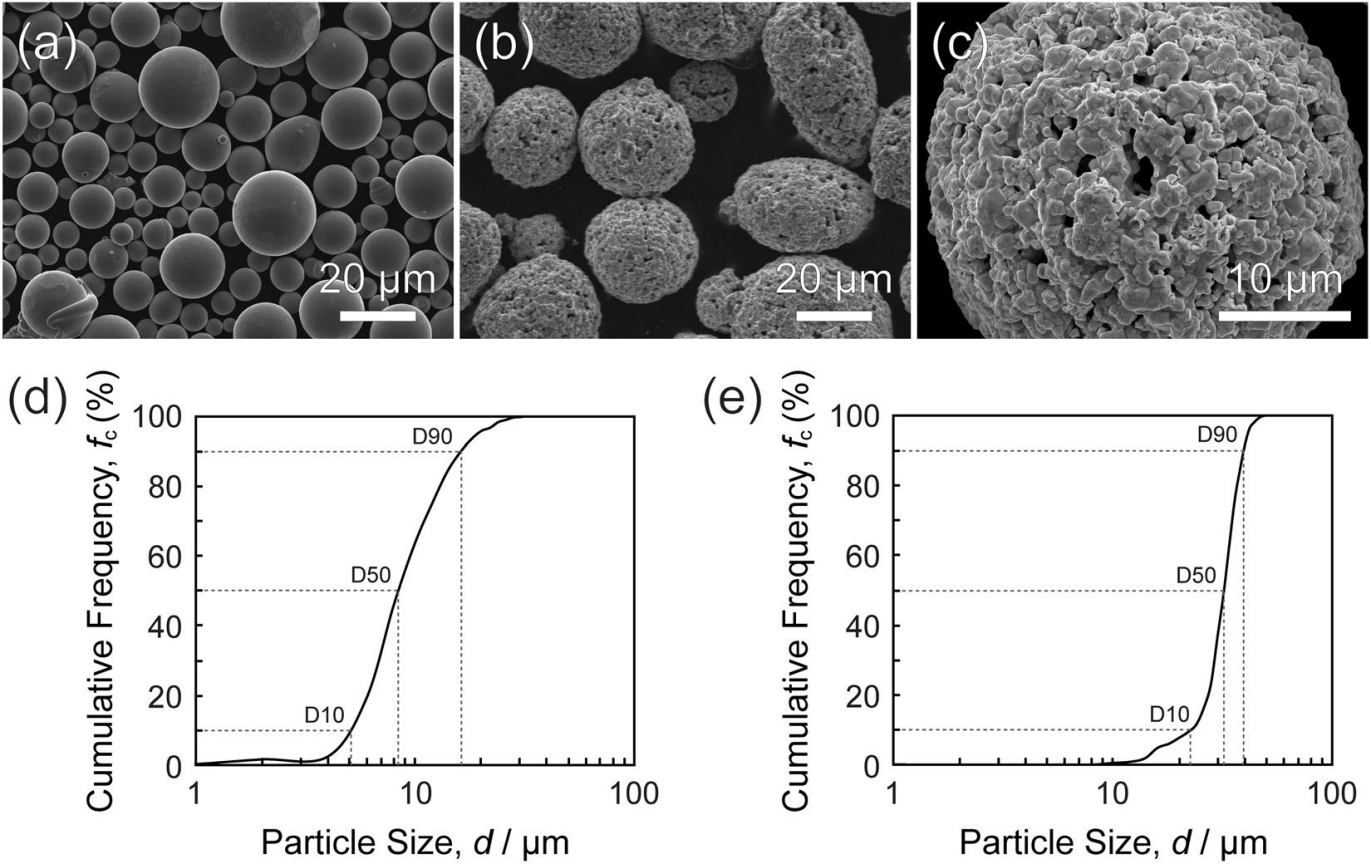
SEM images of the (**a**) Co-Cr-W alloyed powder, (**b**) WC-12 wt.%Co granulated powder, and (**c**) a magnified WC-12 wt.%Co granulated particle with the average WC particle size of 1 μm. Particle size distributions of the (**d**) Co-Cr-W alloyed powder, and (**e**) WC-12 wt.%Co granulated powder.

Co-Cr-W alloyed powder (Sanyo Special Steel Co., Ltd., Hyogo, Japan), which chemical compositions were equivalent to Stellite-6, and WC-12 wt.%Co granulated powder (Japan New Metals Co., Ltd., Osaka, Japan) were selected for investigation. The chemical composition of the Co-Cr-W alloyed powder was shown in Table S1 as supplementary information. These powders were observed by a field emission-scanning electron microscope (FE-SEM: JSM-7100F, JEOL, Tokyo, Japan) at the acceleration voltage of 15 kV. Figure 3(a) to (c) shows SEM images of the (a) Co-Cr-W alloyed powder with spherical morphology, (b) WC-12 wt.%Co granulated powder, and (c) a magnified WC-12 wt.%Co granulated particle with the average WC particle size of 1 μm. Particle size distributions of the (d) Co-Cr-W alloyed powder, and (e) WC-12 wt.%Co granulated powder were determined from SEM images by measuring particle size of 600 particles for each powder. The average particle size, D10, D50, and D90 were evaluated as 10 μm, 5 μm, 8 μm, and 16 μm for the Co-Cr-W alloyed powder and 30 μm, 22 μm, 32 μm, and 40 μm for the WC-12 wt.%Co granulated powder, respectively. C45 carbon steel sheets with 20 mm in length, 20 mm in width and 2.3 mm in thickness were used as substrate.

### Processing

The AM process was performed by an multi-beam LDED system (ALPION Series, Fine-Laser Cladding System, Muratani Machine Inc., Ishikawa, Japan). The system is equipped with six semiconductor lasers with a wavelength of 975 nm and a maximal power output of 60 W (The maximal power output of 300 W in total six lasers). Six oblique-incident lasers were focused on the surface of C45 carbon steel substrate (laser defocus *Δf* of 0 mm) as shown in Fig. 1(b) or 0.5 mm above the substrate (laser defocus *Δf* of 0.5 mm) as shown in Fig. 1(c). Powder containers A and B equipped with powder feeders in the multi-beam LDED system were filled with the Co-Cr-W alloyed powder for the container of A and WC-12 wt.%Co granulated powder for the container of B, respectively. Since the powder feeder was originally designed by Muratani Machine Inc. to realize precision laser cladding, the optimum particle sizes for the feeder were designed in the rage of 20 μm to 75 μm. Powder mixtures having various mixing ratio of the Co-Cr-W alloyed powder and WC-12 wt.%Co granulated powder can be vertically provided on the C45 carbon steel substrate with Ar shielding gas as a carrier from the powder injection nozzle followed by laser melting with controlling powder feeding-rate (PFR) of the powder feeders in the powder containers of A and B, individually. At first, the WC-12 wt.%Co granulated powder were cladded on the substrate by the multi-beam LDED with the laser spot size of 0.3 mm, power output of 140 W, and scan speed of 10 mm/s to investigate the effects of laser defocus *Δf* on single bead morphology. After that, compositionally graded WC/Co-alloy composites were additively manufactured as square shape with 15 mm in length and 15 mm in width by the multi-beam LDED with the laser spot size of 0.3 mm, scan speed of 10 mm/s, pitch width of 0.2 mm, and overlap of 50%. The first layer was made from only Co-Cr-W powder with the PFR of 5 mg/s and power output of 100 W. The second layer was made from the powder mixture of Co-Cr-W alloyed powder (PFR: 5 mg/s) and WC-12 wt.%Co granulated powder (PFR: 15 mg/s) with the power output of 120 W. The third layer was made from only WC-12 wt.%Co granulated powder (PFR: 15 mg/s) with the power output of 120 W. Powder-feeding controllability from the powder injection nozzle of these powders are shown in Fig. S1 as supplementary information. Although the average particle size of the Co-Cr-W alloyed powder was smaller than 20 μm, the powder feeding was well controlled. The other calibration results for PFR are available in our previous literature^16^. The Ar shielding gas flow-rate was set at 0.5 l/min through the experiments.

### Characterization

The cladded beads and composites were observed by a digital microscope (VHX-5000, KEYENCE, Osaka, Japan). Microstructural observations of the beads and composites were performed by the FE-SEM at the acceleration voltage of 15 kV. The chemical compositions of the compositionally graded WC/Co-alloy composites were identified by using an SEM energy-dispersive X-ray spectroscopy (SEM-EDS: X-Max^N^, Oxford Instruments plc, Abingdon, UK). The WC/Co-alloy composites were analyzed by X-ray diffraction (XRD) with an X-ray diffractometer (RINT-2500, Rigaku Corporation, Tokyo, Japan) using Cu-Kα radiation under the operation condition for an accelerating voltage of 40 kV and a current of 200 mA. The diffraction angle 2*θ* was measured from 30° to 120° at a step of 0.01° with a scan speed of 1°/min. Vickers microhardness tests were carried out on the cross-section of the composites with a micro Vickers hardness testing machine (HM-220D, Mitutoyo Corporation, Kawasaki, Japan).

## Results and Discussion

**Figure 4:**
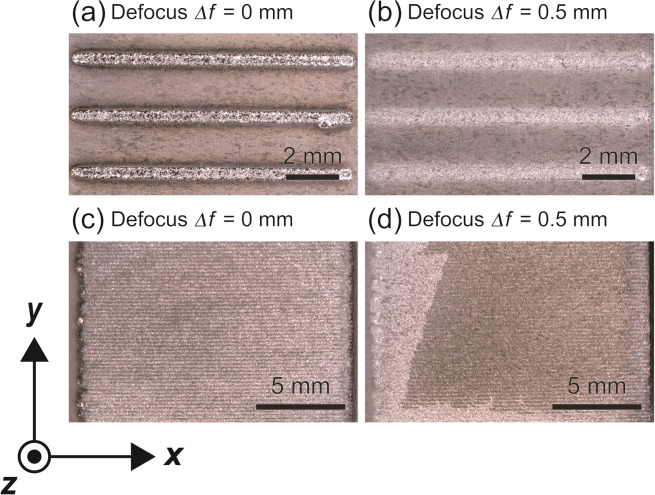
WC-12 wt.%Co beads with the laser defocus *Δf* of (**a**) 0 mm and (**b**) 0.5 mm, and compositionally graded WC/Co-alloy composites consisting of three layers with the laser defocus *Δf* of (**c**) 0 mm^16^ and (**d**) 0.5 mm, additively manufactured on the C45 carbon steel substrate by the multi-beam LDED.

Figure 4 shows WC-12 wt.%Co beads with the laser defocus *Δf* of (a) 0 mm and (b) 0.5 mm, and compositionally graded WC/Co-alloy composites consisting of three layers with the laser defocus *Δf* of (c) 0 mm^16^ and (d) 0.5 mm, additively manufactured on the C45 carbon steel substrate by the multi-beam LDED. In Fig. 4(c,d), the scanned traces can be seen along the *x* direction. These samples were cut along the *y* direction for microstructural observation. The build direction corresponds to the *z* axis.

**Figure 5:**
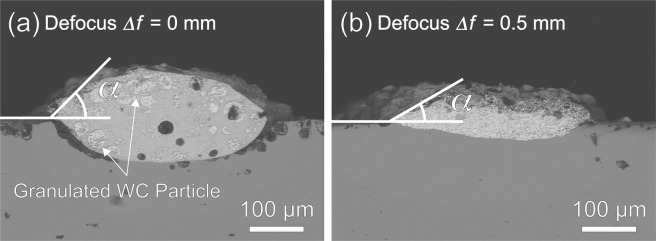
Cross-sectional images (SEM-BEI) of WC-12 wt.%Co single bead cladded on the C45 carbon steel substrate by the multi-beam LDED with the laser defocus *Δf* of (**a**) 0 mm and (**b**) 0.5 mm.

Figure 5(a,b) shows the cross-sectional backscattered electron compositional images (SEM-BEI) of WC-12 wt.%Co single bead cladded on the C45 carbon steel substrate by the multi-beam LDED with the laser defocus *Δf* of (a) 0 mm and (b) 0.5 mm. In Fig. 5(a), it can be seen that some WC-12 wt.%Co granulated particles still keep the initial morphology of aggregates. In addition, a few porosities can be also seen in Fig. 5(a). Therefore, we cannot see the uniformity for *Δf* of 0 mm. On the other hand, the WC-12 wt.%Co granulated particles having the initial morphology of aggregates cannot be seen in the bead for *Δf* of 0.5 mm in Fig. 5(b). The bead area became smaller for *Δf* of 0.5 mm than 0 mm due to the change of processing mode from melt-pool mode to thermal spray mode (Details of this change of processing mode will be discussed in the last section). The contacting angles α of the beads were 45° for *Δf* of 0 mm and 30° for *Δf* of 0.5 mm. These results imply that the multi-beam LDED process was performed at higher temperature for the laser defocus *Δf* of 0.5 mm than that of 0 mm. Since the melting point of Co bond is 1495 °C, it is suggested that the multi-beam LDED process with the laser defocus *Δf* of 0.5 mm was carried out at over 1495 °C.

**Figure 6:**
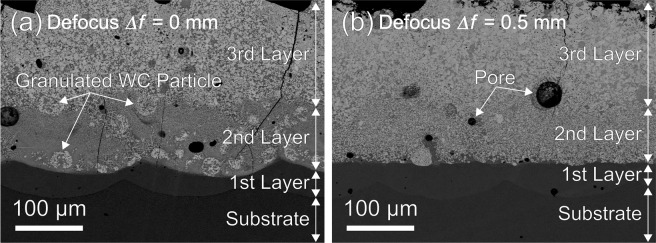
Cross-sectional images (SEM-BEI) of the compositionally graded WC/Co-alloy composites additively manufactured on the C45 carbon steel substrate by the multi-beam LDED with the laser defocus *Δf* of (**a**) 0 mm and (**b**) 0.5 mm.

Figure 6(a,b) shows the cross-sectional images (SEM-BEI) of the compositionally graded WC/Co-alloy composites additively manufactured on the C45 carbon steel substrate by the multi-beam LDED with the laser defocus *Δf* of (a) 0 mm and (b) 0.5 mm. The first layer was made from Co-Cr-W powder with the PFR of 5 mg/s. The second layer was made from the powder mixture of Co-Cr-W alloyed powder (PFR: 5 mg/s) and granulated WC-12 wt.%Co powder (PFR: 15 mg/s). The third layer was made from granulated WC-12 wt.%Co powder (PFR: 15 mg/s). Although cracks and pores can be seen in the multi-layered composites, the compositionally graded WC/Co-alloy composites were successfully obtained as shown in Fig. 6(a,b). It should be noted that the most significant difference caused by the laser defocus *Δf* condition is dispersion state of the WC-12 wt.%Co granulated powder in the second layer. In Fig. 6(a), some WC-12 wt.%Co granulated particles were dispersed as the initial morphology of aggregates. This dispersion state is similar to the one shown in Fig. 1(a). In contrast, the initial WC-12 wt.%Co granulated particles cannot be seen in Fig. 6(b). For the laser defocus *Δf* of 0.5 mm, the WC particles were homogeneously dispersed in the second layer as the dispersion state shown in Fig. 1(c). This results means that the Co bond in the WC-12 wt.%Co granulated particles was completely melt by the multi-beam LDED. These experimental observations also imply that the multi-beam LDED processing was performed at higher temperature for the laser defocus *Δf* of 0.5 mm than that of 0 mm. Therefore, it was demonstrated that the multi-beam LDED processing temperature can be controlled by changing the laser defocus *Δf*.

**Figure 7:**
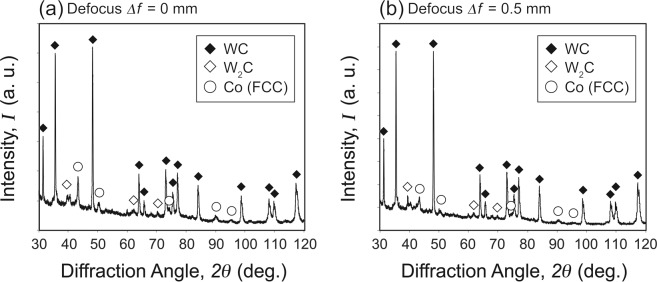
XRD profiles for the third layer of compositionally graded WC/Co-alloy composites additively manufactured by the multi-beam LDED with laser defocus *Δf* of (**a**) 0 mm and (**b**) 0.5 mm.

Figure 7(a,b) shows the XRD profiles for the third layer of compositionally graded WC/Co-alloy composites additively manufactured by the multi-beam LDED with laser defocus *Δf* of (a) 0 mm and (b) 0.5 mm. In Fig. 7(a,b), main peaks corresponded to WC. Although W_2_C peaks was also identified in the XRD profile due to the decarburization, intensity of W_2_C peaks were very low. Other phases such as eta phase (M_6_C), and M_7_C_3_ were not detected by the XRD. The peaks for Co with FCC phase originated from bond material was observed as well. This Co with FCC phase often observed after processing of fine particles at higher temperature^17,18^, possibly due to the solid solution of W into the Co phase or the size-dependent crystal phase of Co^19^. Since the formation of W_2_C, M_6_C, and other phases such as M_7_C_3_ lead to brittleness of the composites^20^, the performed multi-beam LDED processing condition is appropriate for the intended purposes of coating. No significant difference was confirmed between the two samples with laser defocus *Δf* of 0 mm and 0.5 mm in terms of the XRD measurements.

**Figure 8:**
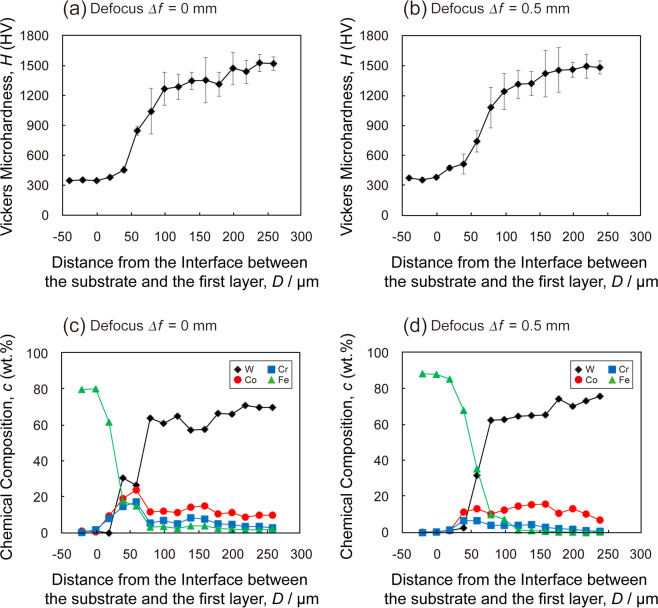
Vickers microhardness with the laser defocus *Δf* of (**a**) 0 mm and (**b**) 0.5 mm, and chemical compositions with the laser defocus *Δf* of (**c**) 0 mm and (**d**) 0.5 mm, as a function of distance from the interface between the substrate and the first layer of compositionally graded WC/Co-alloy composites additively manufactured by the multi-beam LDED.

Figure 8 shows the Vickers microhardness *H* with the laser defocus *Δf* of (a) 0 mm and (b) 0.5 mm, and chemical compositions *c* with the laser defocus *Δf* of (c) 0 mm and (d) 0.5 mm, as a function of distance from the interface between the substrate and the first layer of compositionally graded WC/Co-alloy composites additively manufactured by the multi-beam LDED. Here, error bars indicate the standard deviation. The Vickers microhardness *H* increased from 350 HV to 1530 HV for *Δf* of 0 mm, and from 380 HV to 1500 HV for *Δf* of 0.5 mm. The variation of the Vickers microhardness *H* has a strong correlation with that of the chemical composition of W. Since almost initial WC particles were identified as WC in the third layer by the XRD, the variation of the Vickers microhardness *H* is mainly dominated by the volume fraction and distribution of WC. The concentration of Fe (the substrate material) in the third layer was 2% for *Δf* of 0 mm, and 0.3% for *Δf* of 0.5 mm. It can be understood that diffusion occurred due to the melt pool mode for *Δf* of 0 mm. Meanwhile, it is suggested that diffusion could be suppressed due to the thermal spray mode for *Δf* of 0.5 mm. The preferable property gradients in the WC/Co-alloy composites could be made with the multi-beam LDED system by controlling the feeding rate of the powders and laser-beam defocus *Δf*.

**Figure 9:**
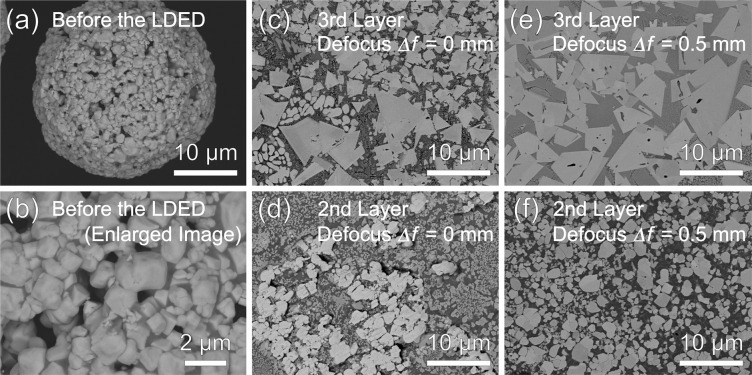
(**a**) A granulated WC-12 wt.%Co particle with the average WC particle size of 1 μm, and (**b**) an enlarged image of (**a**). Microstructures of (**c**) the third and (**d**) second layer for *Δf* of 0 mm, and (**e**) the third and (**f**) second layer for *Δf* of 0.5 mm in compositionally graded WC/Co-alloy composites.

**Figure 10:**
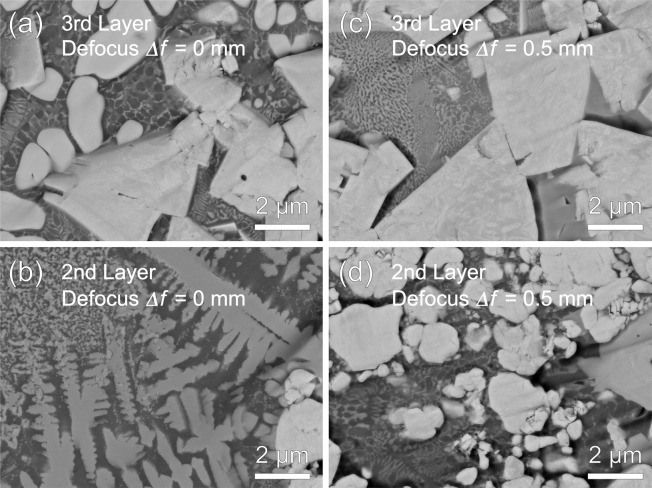
Magnified images of microstructures of (**a**) the third and (**b**) second layer for *Δf* of 0 mm, and (**c**) the third and (**d**) second layer for *Δf* of 0.5 mm in compositionally graded WC/Co-alloy composites.

Figure 9 shows (a) an initial WC-12 wt.%Co granulated particle with the average WC particle size of 1 μm, and (b) an enlarged image of (a). Microstructures of the second and third layer for *Δf* of 0 mm and 0.5 mm in compositionally graded WC/Co-alloy composites are also shown in Fig. 9(c) to (f). Average WC particle size in the second and third layer are measured as 1.5 μm and 2.2 μm for *Δf* of 0 mm, and 1.4 μm and 5.9 μm for *Δf* of 0.5 mm. Since some WC particles reacted with the Co and Co-Cr-W alloy and formed M_6_C in the second layer for *Δf* of 0 mm, the some gray-colored particles were smaller than the ones in the initial WC-12 wt.%Co granulated particle as shown in Fig. 9(d). Therefore, these gray-colored particles were ignored for WC particle size measurement. Grain growth of the WC particles can be observed in Fig. 9(c–f). The WC particle sizes in the third layer for *Δf* of 0.5 mm are larger than the ones for *Δf* of 0 mm. Especially, liquid phase sintering followed by particle rearrangement can be observed in the third layer for *Δf* of both 0 mm and 0.5 mm as the faceted particles can be confirmed^21^. Allibert investigated microstructure of the liquid-phase sintered WC-8 wt.%Co with initial average WC particle size of 0.85 µm at 1450 °C for 8 h^22^, and obtained a similar microstructure having almost the same WC particle size shown in Fig. 9(e). Therefore, the grain growth of WC particles occurs much faster in the multi-beam LDED process compared with the conventional sintering process. In addition, the grain growth of WC was more accelerated by changing the laser defocus *Δf* from 0 mm (the melt pool mode) to 0.5 mm (the thermal spray mode) as the powder stream were directly heated with focused six lasers by suppressing thermal conduction between powder and substrate. Another reason for this accelerated grain growth of WC by the laser defocusing was explained in the next paragraph. Figure 10 shows magnified images of microstructures of (a) the third and (b) second layer for *Δf* of 0 mm, and (c) the third and (d) second layer for *Δf* of 0.5 mm. In metal matrix of the third layer, equiaxed structure of primary phase with average size of 300 nm surrounded by eutectic phase can be seen for *Δf* of 0 mm, whereas finer cellular structure consisting of primary and eutectic phase was formed for *Δf* of 0.5 mm. A significant difference was observed in the second layer. Although a lot of eta phase with particle shape and dendritic shape, which is identified by the SEM-EDS, was observed for *Δf* of 0 mm, WC particles were mainly formed for *Δf* of 0.5 mm.

**Figure 11:**
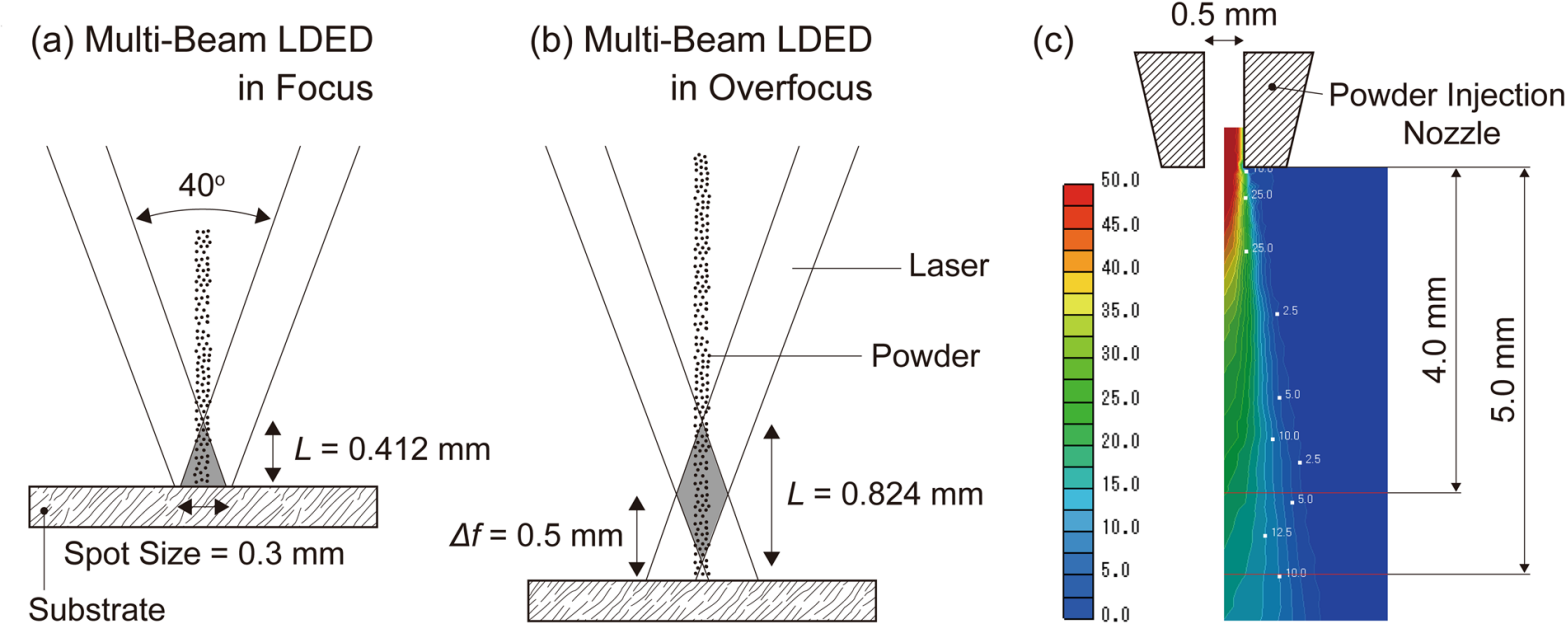
Schematic illustrations showing laser geometry and particle travelling distance during the laser focusing area in the multi-beam LDED system in (**a**) focus and (**b**) overfocus of multi lasers on the surface of the substrate. (**c**) Velocity map of particle vertically injected from the powder injection nozzle. This map was obtained by simulation with SCRYU/Tetra V14 software.

In order to obtain WC/Co-alloy composites with uniformly dispersed WC particles by the LDED, the melting of Co bond in the WC-12 wt.%Co granulated particles is crucial as shown in Figs. 6 and 9. The effects of laser defocus *Δf* on temperature increase of flying powder during the laser focusing area were estimated. The energy required to heat and melt the particle can be written as

1 $$\pi {r}_{{\rm{p}}}^{2}P{\alpha }_{{\rm{L}}}t=\frac{4}{3}\pi {r}_{{\rm{p}}}^{3}\rho [{C}_{{\rm{p}}}(T-{T}_{{\rm{a}}})+\Delta {H}_{{\rm{m}}}],$$

where *r*_p_ is the radius of particle, *P* the laser power density, *α*_L_ the laser absorption of particle, *t* the particle travelling time during the laser focusing area, *ρ* the mass density of particle, *C*_p_ specific heat capacity at constant pressure, *T* temperature of particle at exit position of the laser focusing area, *T*_a_ ambient temperature, Δ*H*_m_ latent heat of melting^23^. As the average particle size of the WC-12 wt.%Co granulated particles is 30 μm, the value of *r*_p_ is 15 μm. Using the power output of 120 W and the laser spot size of 0.3 mm, the laser power density *P* becomes about 1700 W/mm^2^. Since the laser absorption of WC and Co are 0.82 and 0.58^24^, respectively, the laser absorption *α*_L_ of the WC-12 wt.%Co granulated particle is 0.77 by considering the volume fraction of Co as 19.3%. In the same way, the mass density of the WC-12 wt.%Co granulated particle is estimated as 7.0 × 10^−3^ g/mm^3^ by considering the volume fraction of Co as 19.3% and the presence of large pores as 50% due to the granulated particle^24^. The specific heat capacity *C*_p_ of the WC-12 wt.%Co granulated particle is 0.2 J/gK^25^. The latent heat of melting Δ*H*_m_ is ignored in this estimation. Figure 11 shows schematic illustrations showing laser geometry and particle travelling distance during the laser focusing area in the multi-beam LDED system in (a) focus and (b) overfocus of multi lasers on the surface of the substrate. The angle between two laser beams facing each other directly are 40° in this LDED system. Since the spot size was set as 0.3 mm, particle travelling distance *L* in the laser focusing area, shaded area in Fig. 11(a,b), became 0.412 mm for *Δf* of 0 mm and 0.824 mm for *Δf* of 0.5 mm. Figure 11(c) shows velocity map of particle vertically injected from the powder injection nozzle. This map was obtained by simulation with SCRYU/Tetra V14 software. The position of 4.0 mm directly below the powder injection nozzle corresponds to the entry position of the laser focusing area in Fig. 11(a,b). Average particle velocities during the laser focusing area were estimated as 19 m/s for *Δf* of 0 mm and 18 m/s for *Δf* of 0.5 mm. Thus, the particle travelling times *t* during the laser focusing area were estimated as 2.2 × 10^−5^ s for *Δf* of 0 mm and 4.6 × 10^−5^ s for *Δf* of 0.5 mm, respectively. Finally, the temperature *T* of particle at exit position of the laser focusing area were estimated as 980 °C for *Δf* of 0 mm and 2050 °C for *Δf* of 0.5 mm with the ambient temperature *T*_a_ of 20 °C. Consequently, the WC-12 wt.%Co granulated particles get in the melt pool with the initial granulated particle shape for *Δf* of 0 mm since the melting temperature *T*_m_ of cobalt is 1495 °C (the melt pool mode). In contrast, it should be noted that the Co bond in the WC-12 wt.%Co granulated particles can be completely melt during the laser focusing area for *Δf* of 0.5 mm (the thermal spray mode). These results also support the reason for the accelerated grain growth of WC by the laser defocusing. These experimental results and analyses demonstrated the effectiveness of the laser-beam-defocus function in the multi-beam LDED system as a key factor for tailoring microstructure of additively manufactured MMCs, and indicated the potential of the multi-beam LDED to obtain functionally graded MMCs with uniformly-dispersed reinforcements for advanced functional coatings with high qualities.

## Conclusions

AM process of compositionally graded WC/Co-alloy composites on the C45 carbon steel substrate by the multi-beam LDED system was investigated. For tailoring microstructures of compositionally graded WC/Co-alloy composites with uniformly-dispersed reinforcements, the combinational method: the laser-beam defocus function in the multi-beam LDED system and granulated powder was attempted. The preferable property gradients in the WC/Co-alloy composites could be made with the multi-beam LDED system by controlling the feeding rate of the powders and laser-beam defocus *Δf*. The findings can be summarized as follows.

(1) By laser defocusing in the multi-beam LDED system, the bead area and contacting angles α of the beads became smaller for *Δf* of 0.5 mm than 0 mm due to the change of processing mode from melt-pool mode to thermal spray mode. These results imply that the multi-beam LDED processing was performed at higher temperature for the laser defocus *Δf* of 0.5 mm than that of 0 mm.

(2) By laser defocusing in the multi-beam LDED system, the Co bond in the WC-12 wt.%Co granulated particles was completely melt. This results led to the homogeneous microstructures of compositionally graded WC/Co-alloy composites with uniformly-dispersed reinforcements by the AM process.

(3) The grain growth of WC was more accelerated by changing the laser defocus *Δf* from 0 mm (the melt pool mode) to 0.5 mm (the thermal spray mode) as the powder stream were directly heated with focused six lasers by suppressing thermal conduction between powders and the substrate.

## Supplementary information

Supplementary Information.
