# Supplementary material for: Effects of Laser-Beam Defocus on Microstructural Features of Compositionally Graded WC/Co-Alloy Composites Additively Manufactured by Multi-Beam Laser Directed Energy Deposition
Source: Sci Rep. 2020 Jun 2;10:8975. doi: 10.1038/s41598-020-65429-8 (PMC7265354; doi:10.1038/s41598-020-65429-8)
Supplement: Supplementary file 1 — Supplementary Information. [file 41598_2020_65429_MOESM1_ESM.pdf]

Supplementary File

Effects of Laser-Beam Defocus on Microstructural Features of Compositionally Graded WC/Co-Alloy Composites Additively Manufactured by Multi-Beam Laser Directed Energy Deposition

Authors: Takahiro Kunimine, Ryusei Miyazaki, Yorihiro Yamashita, and Yoshinori Funada

Table S1. Chemical composition of Co-Cr-W alloyed powder (in wt.%).

C	Si	Ni	Cr	W	Co	Fe
1.277	1.39	1.30	30.15	4.65	Bal.	0.82

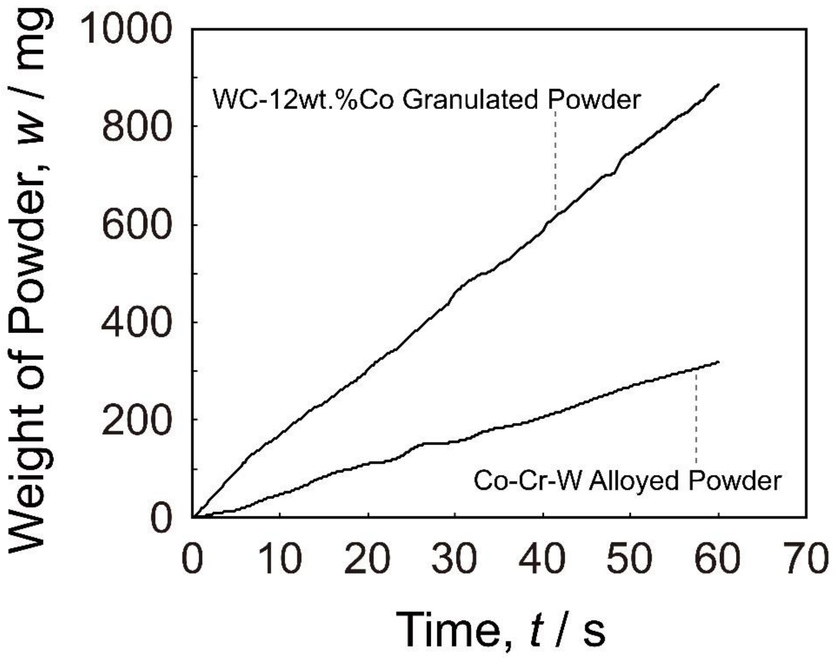

Figure S1. Weight of powders supplied from the powder injection nozzle as a function of time.
